# Influenza Virus: A Master Tactician in Innate Immune Evasion and Novel Therapeutic Interventions

**DOI:** 10.3389/fimmu.2018.00743

**Published:** 2018-04-12

**Authors:** Alan Chen-Yu Hsu

**Affiliations:** ^1^Viruses, Infections/Immunity, Vaccines & Asthma, Hunter Medical Research Institute, Newcastle, NSW, Australia; ^2^Priority Research Centre for Healthy Lungs, Faculty of Health and Medicine, The University of Newcastle, Newcastle, NSW, Australia

**Keywords:** influenza, influenza A virus, virulence factors, hemagglutinin, polymerase acidic 1-F2, NS1, therapeutics

## Abstract

Influenza is a contagion that has plagued mankind for many decades, and continues to pose concerns every year, with millions of infections globally. The frequent mutations and recombination of the influenza A virus (IAV) cast a looming threat that antigenically novel strains/subtypes will rise with unpredictable pathogenicity and fear of it evolving into a pandemic strain. There have been four major influenza pandemics, since the beginning of twentieth century, with the great 1918 pandemic being the most severe, killing more than 50 million people worldwide. The mechanisms of IAV infection, host immune responses, and how viruses evade from such defensive responses at the molecular and structural levels have been greatly investigated in the past 30 years. While this has advanced our understanding of virus–host interactions and human immunology, and has led to the development of several antiviral drugs, they have minimal impact on the clinical outcomes of infection. The heavy use of these drugs has also imposed selective pressure on IAV to evolve and develop resistance. Vaccination remains the cornerstone of public health efforts to protect against influenza; however, rapid mass-production of sufficient vaccines is unlikely to occur immediately after the beginning of a pandemic. This, therefore, requires novel therapeutic strategies against this continually emerging infectious virus with higher specificity and cross-reactivity against multiple strains/subtypes of IAVs. This review discusses essential virulence factors of IAVs that determine sustainable human-to-human transmission, the mechanisms of viral hijacking of host cells and subversion of host innate immune responses, and novel therapeutic interventions that demonstrate promising antiviral properties against IAV. This hopefully will promote discussions and investigations on novel avenues of prevention and treatment strategies of influenza, that are effective and cross-protective against multiple strains/subtypes of IAV, in preparation for the advent of future IAVs and pandemics.

## Introduction

Influenza A viruses (IAVs) and their continuous emergence/re-emergence are undoubtedly an important cause of global concern, morbidity and mortality, clinical, and socio-economical burden in humans. The emergence of highly pathogenic avian influenza virus H5N1 in 1997 and 2003, H7N7 in 2003, recent influenza H1N1 pandemic in 2009 (H1N1pdm09), and H7N9 in 2013 have caused tremendous mortality in the affected populations, and in our current inter-connected world, the concerns and impact of potential pandemics are truly global.

The ongoing circulation of IAVs in their natural hosts and the ever-mutating antigenicity frequently leads to the appearance of novel viruses with the ability to cross species barriers and infect humans with unpredictable pathogenicity. Furthermore, effective immuno-evasive and modulatory strategies employed by IAVs have made it difficult for both the innate and acquired immunity to combat the infections without the help of vaccination.

The early innate immune responses to IAV infections are essential in the immediate control of viral replication and spread. However, IAVs have also evolved to produce only a handful, but multi-functional proteins to combat the intricate layers of human innate immune signaling pathways. These virulence factors inhibit host antiviral immunity, while stimulating inflammatory responses in the airways (cytokine storm) that cause severe symptoms. It is, therefore, imperative to understand the molecular mechanisms of IAV-mediated modulation of innate immunity by these viral factors in order to identify potential therapeutic options.

While antiviral drugs are mostly used either prophylactically or in treatment, they have minimal impact on the course of the disease. Heavy reliance on antiviral drugs has also placed a strong selective pressure on IAVs to mutate and develop resistance (1, 2). The unpredictable nature of novel IAVs and limited prevention and treatment options, therefore, urges the research and development of novel approaches against IAVs. This review summarizes the current understanding of the mechanisms of IAV infection in humans, the ways by which IAV suppresses antiviral immunity and causes the inflammatory cytokine storm, and novel peptide-based anti-influenza drugs that may potentially be beneficial as preventative and treatment strategies in current and future IAV pandemics.

## IAV—Simple and Elegant

**Figure 1:**
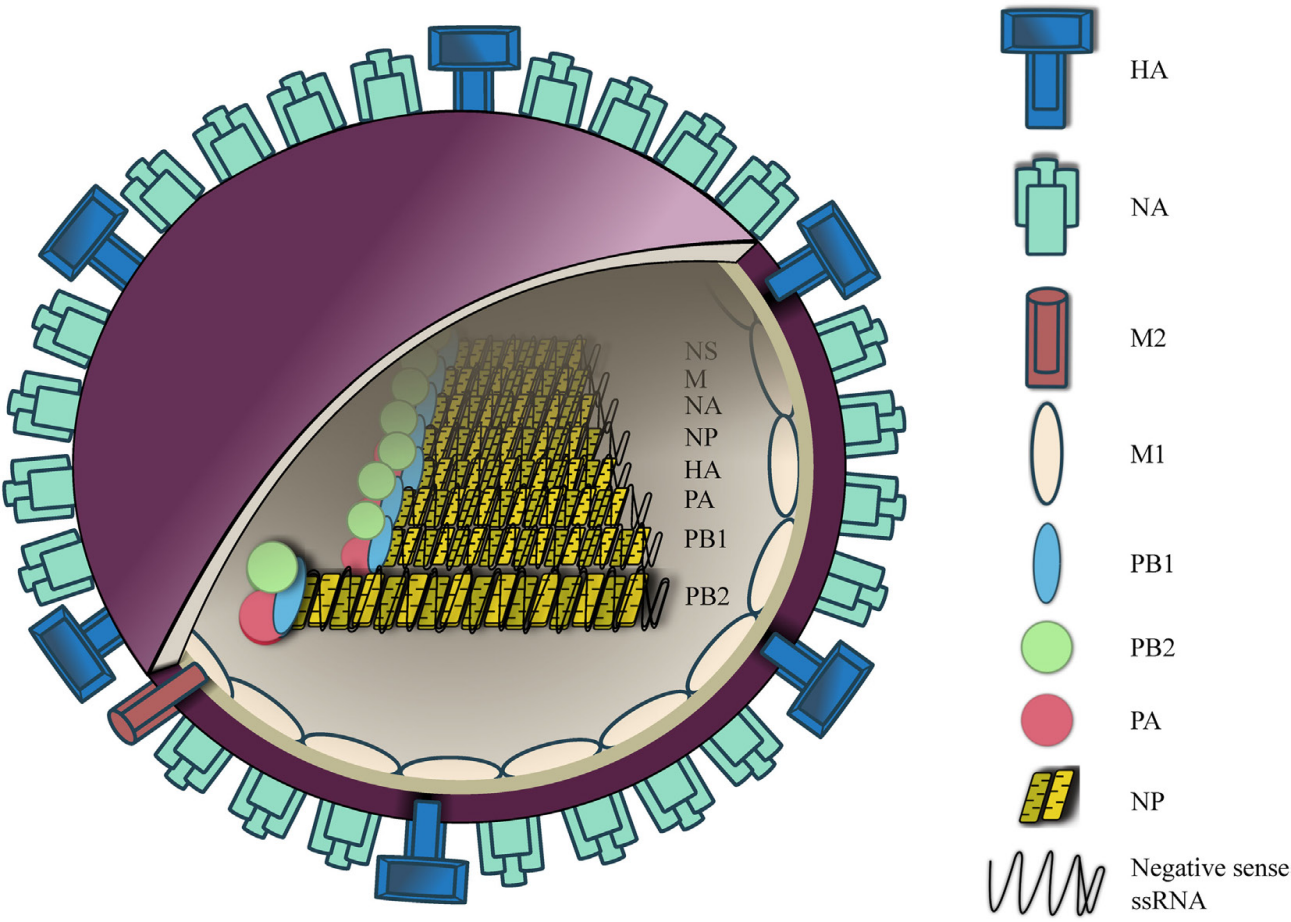
Influenza A virus (IAV) structure. IAV contains an outer membrane envelope with hemaglutinin (HA), neuraminidase (NA), and matrix 2 (M2) ion channels, and an inner M1 protein layer that encloses an eight segmented RNA genome. The RNA segments are folded into a helical hairpin structure with nucleoprotein, which are complexed with heterotrimeric RNA-dependent RNA polymerase [polymerase basic (PB1), PB2, and polymerase acidic (PA)].

IAVs is a negative sense, single-stranded RNA virus (~80–120 nm in diameter) (3). The viral envelope features two surface glycoproteins, hemaglutinin (HA) and neuraminidase (NA), at a ratio of four HA to one NA (4). A small number of M2 ion channel is also embedded in the viral envelope at a ratio of one M2 channel to 10^1^–10^2^ HA molecules (5) (Figure 1).

Within the envelope are the eight segments of influenza RNA, which are presented in a form of helical hairpin structure, each of which is coated with arginine-rich nucleoprotein (NP) (6–8). NP-RNA is complexed with heterotrimeric RNA-dependent RNA polymerase, which consists of three subunits, polymerase basic (PB)1, PB2, and polymerase acidic (PA) (3). Segment 1, 3, 4, 5, and 6 encodes for PB2, PA, HA, NP, and NA, respectively. Segment 2 encodes for PB1 and *via* a different reading frame an accessory protein PB1-F2 is expressed in most influenza A strains. Segment 7 encodes for matrix 1 (M1) protein and also M2 ion channel *via* alternative splicing. Segment 8 encodes for non-structural protein (NS)-1 and nuclear export protein. At both 3′ and 5′ ends of each segment lies a non-coding region of varying length that acts as a promoter site for viral polymerase complex to initiate transcription. This region also contains an mRNA polyadenylation signal and a signal for virus assembly.

The primary site of infection by IAVs are epithelial cells of the respiratory mucosa. Airway epithelial cells are both susceptible and permissive to IAV infection. This occurs by the binding of HA to the sialyl sugar chain receptors on the host cell surface, allowing the virus to be internalized into endosomes in the host epithelial cells. The low pH environment of the endosome promotes HA to undergo conformational change that liberates and inserts a fusion peptide from the amino terminus of HA into the endosomal membrane. This spring-loaded mechanism fuses the viral envelope and the membrane together, thereby releasing viral RNP into the host cytoplasm (9, 10). The M2 ion channel also allows an influx of H^+^ ions into the virion, and lowers the intra-virionic pH. This in turn disrupts the viral RNP-M1 protein interaction and subsequently releases viral RNP into host cellular cytoplasm (11–14). Released IAV RNAs and polymerases are then translocated into the nucleus where viral replication occurs. The newly synthesized viral structural proteins and viral segments then traffic to lipid rafts on the plasma membrane to be released (15, 16). Since the viral envelope is derived from the host membrane, which contains sialic acid glycoproteins, the newly formed virion remains intact on the host cell surface. The viral NA cleaves the host cell surface sialic acid residues, releasing the newly formed virions free from the host cell surface.

## HA—Structurally Plastic and Absolutely Essential for Infectivity, Human Transmission, and Pandemic

IAVs HA is responsible for the entry of the virus in the host cells by binding to host cell surface glycoproteins terminated with sialic acid residues at specific linkages. Human IAVs preferentially bind to glycoproteins containing the terminal SAα2,6Gal linkage, which are predominately found in human upper airway epithelium (17–19). In contrast, avian IAVs bind to that with terminal SAα2,3Gal linkage in the lower airways (17–21). This difference in binding specificity and distribution of sialic acid residues may in part explain why highly pathogenic avian influenza virus H5N1 is currently incapable of transmitting from human to human in a sustainable manner. In contrast, pig trachea contains both SAα2,6Gal and SAα2,3Gal linkages, indicating that pigs can act as an intermediate mixing host (22–24), allowing the reassortment of both avian and human viruses to occur. The prime example is the emergence of the 2009 H1N1 pandemic, which was the result of triple reassortment between avian, swine, and human IAVs in pigs (25, 26). The difference in their binding specificity can be explained by the amino acid residue at position 226 of HA glycoprotein. HA of human influenza viruses contains a Leu226 that results in the preferential binding to SAα2,6Gal linkage. In contrast, HA of avian influenza viruses have a Gln226, which binds to SAα2,3Gal-linked glycoproteins (27, 28).

Two recent important investigations further characterized the molecular changes that are required for H5N1 to become transmissible among humans. Imai et al. showed that Gln226Leu/Gly228Ser increased the binding of H5 HA to SAα2,6Gal while retaining the binding capacity to SAα2,3Gal. When Asn158Asp/Asn224Lys/Thr318Ile was introduced with Gln226Leu/Gly228Ser, this fully converted the virus with H5 HA in an H1N1pdm09 backbone to achieve sustainable aerosol transmission in a ferret model (29). In addition, Herfst et al. also demonstrated that mutant H5N1 containing His103Tyr, Thr156Ala, and Gln222Leu in the HA protein, and Glu627Lys in the viral polymerase protein PB2 was able to efficiently transmit between ferret models *via* aerosols (30). Interestingly, although not surprising, most of these residues (Asn158Asp; Gln222Leu; Asn224Lys; Gln226Leu; Gly228Ser) are at the sialic acid residue binding site of the globular domain.

**Figure 2:**
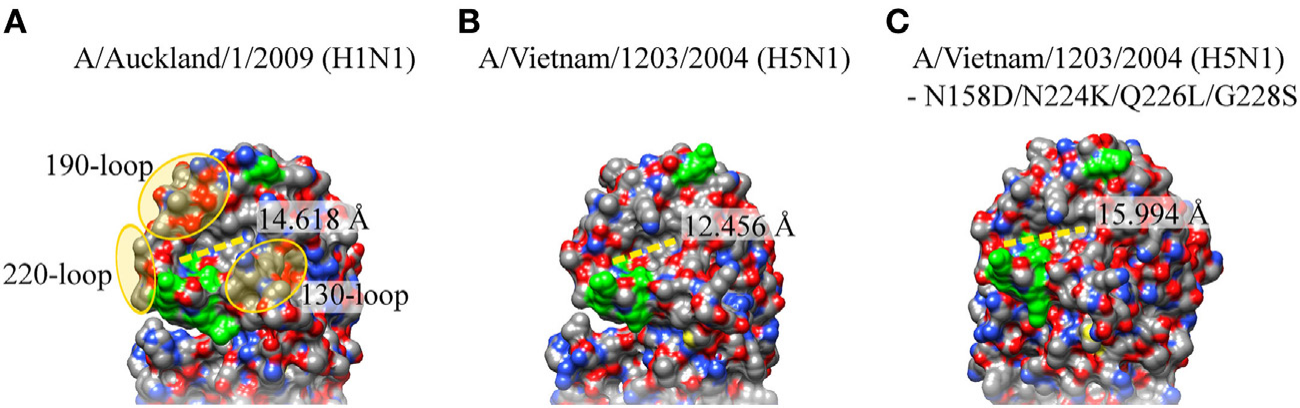
Crystal structure of hemaglutinin (HA) sialic acid residue binding site of H1N1pdm09, H5N1, and airborne-transmissible H5N1. The sialic acid residue binding site on the HA of **(A)** A/Washington/5/2011 (H1N1pdm09; PDB: 4LKX), **(B)** A/Vietnam/1203/2004 (H5N1; PDB: 2FK0), and **(C)** H5N1 with Asn148Asp, Gln222Leu Asn224Lys Gln226Leu, and Gly228Ser. These amino acid residues were changed *in silico* and modeled using HA of H5N1(PDB: 2FK0) as a template in SWISS-MODEL. Protein visualization and distance calculation was performed in UCSF Chimera molecular visualization application.

When these amino acid residues in H5N1 HA were changed *in silico* to the ones in the airborne-capable HA, and structurally modelled using the wild-type H5N1 HA as a template in SWISS-MODEL (31), the binding cavity in the mutant, surrounded by the 130-, 190-, and 220-loop, may potentially be lengthened and result in a slightly larger binding cavity (Figure 2). Furthermore, Asn158Asp is also likely to make the receptor binding pocket slightly more acidic, further facilitating a more efficient airborne transmission. A mutant H1N1 with Tyr17His (pH 6.0) in HA showed loss of airborne transmission, and was less efficient in contact transmission with reduced pathogenicity in mice, and His17Tyr (pH 5.3) revertant virus regained the airborne transmissibility and pathogenicity (32). This may also widen the binding capacity toward moieties with other linkages/modifications. While changes in these residues are important in airborne transmission in ferret models, it remains unclear whether similar or other changes in HA need to occur for a sustainable airborne transmission in humans, these studies provide valuable insight into cause of efficient human transmission, surveillance for potential human transmissible IAVs, and new approach into therapeutic designs.

Epidermal growth factor receptor, a glycoprotein with potential terminal SAα2,6Gal and SAα2,3Gal linkages (33, 34), has been shown to be important in IAV entry into host cells in a ligand (EGF)-independent, and phosphoinositide-3 kinase (PI3K)-activation-dependent manner (35–37). However, IAV has also been demonstrated to cause infection in the absence of its respective receptors. H3N2 and H1N1 was able to infect and replicate to the similar titer in the lung of the mice lacking receptors with SAα2,6Gal linkages compared to wild type mice (38). Consistent with this finding, while human bronchial epithelial cells showed higher levels of SAα2,6Gal compared with SAα2,3Gal linkages, human IAV H3N2, and a low pathogenic avian H11N9 have been shown to enter into bronchial epithelial cells at a similar rate (39). This indicates that sialic acid residues may not be absolutely essential in influenza virus entry, and other moieties, such as sulfonated or fucosylated glycoproteins may also be targeted by HA protein (40, 41).

## The Polymerase Constellation

IAV RNA is always packaged in a heterotrimeric RNA-dependent RNA polymerase complex with viral polymerase basic (PB)1, PB2, polymerase acidic (PA), and nucleoprotein (NP), and upon successful entry into epithelial cells, this complex translocates to the nucleus where replication occurs. As IAV viral RNA does not contain a 5′ cap as they do on host RNAs, PB2 contains a cap-binding domain that binds to the 5′ cap of nascent host RNAs, and PA then “steals” the cap off the host RNA by cleavage, which is then used as primers to initiate transcription of viral mRNAs. This process is called cap-snatching. This polymerase complex, therefore, is critical in the survival of IAVs, and has been implicated in the host range determination. Clements et al. demonstrated that the reassortment virus containing the PB2 gene of avian origin and all other genes from human IAV is able to replicate efficiently in the avian host, while showing restricted replication in the mammalian respiratory tract (42). This reassortment virus was then progressively passaged to generate mutant viruses that were able to replicate in mammalian tissues. Nucleotide sequence analysis revealed that this phenotype of the reassortment virus was due to the Glu627 of the PB2 gene (43). A single amino acid substitution to a lysine residue allowed this mutant reassortment virus to replicate efficiently with increased pathogenicity in the mammalian system (44–47). All the avian IAVs analyzed to date have a Glu627 in the PB2, whereas human IAVs have a Lys627, indicating this residue at position 627 of PB2 is an important host range determinant of IAVs (43). Furthermore, Glu627Lys substitution being a prerequisite for aerosol to airborne transmissibility switch is troubling, as the viruses isolated from the recent fatal cases of H7N9 infection in China carried Glu627Lys substitution, but the viruses isolated from poultry did not (48, 49).

The compatibility of other subunits of the polymerase complex is also involved in the host range specificity (50, 51). Reassortment viruses with human PA and avian PB1 and PB2, or with human PA and PB2 and avian PB1 showed a restricted viral replication in mammalian cells, indicating that a specific constellation of polymerase genes may be involved in the host range specificities (51).

## PB1-F2—A Pyrogenic Death Dealer

In the middle of searching for an IAV-derived out-of-frame polypeptide that can be recognized by CD8^+^ T lymphocytes, an 11th gene, PB1-F2, was serendipitously discovered (52). PB1-F2 is an accessory protein that is translated from an alternative +1 open reading frame of PB1. It modulates innate immune responses at multiple signaling levels, leading to increased inflammatory and impaired antiviral responses. PB1-F2 contains a mitochondrial targeting sequence at the carboxyl-terminus and has a pro-apoptotic effect on immune cells (53). By binding to the inner and outer mitochondrial membrane transport protein adenine nucleotide translocator 3 and voltage-dependent anion channel 1 (VDAC1), PB1-F2 disrupts mitochondrial integrity, releasing cytochrome C, and leading to apoptosis. Another study also demonstrated that PB1-F2 can be translocated into the inner mitochondrial space in a TOM40-dependent manner, and impair mitochondrial function (54).

Host innate immune response is triggered by the pattern recognition receptors including retinoic acid inducible gene 1 (RIG-I). RIG-I binds to IAV RNAs and forms a complex with the adaptor protein tripartite motif-containing protein (TRIM)25 (55). This complex interacts with mitochondrial antiviral signaling (MAVS) protein to induce the activation of interferon (IFN)-regulatory factor (IRF) 3, which initiates the induction of important antiviral cytokines including type I and III IFNs (56, 57). These IFNs then stimulate the expression of over 300 IFN-stimulated genes (ISGs) such as protein kinase R (PKR) to limit viral replication (58). Toll-like receptor 3 also recognizes IAV RNAs and activates an important transcription factor nuclear-factor-kappa-B (NF-κB), which stimulates the expression of predominantly inflammatory cytokines, including interleukin (IL)-6 and IL-1β (59–61).

PB1-F2 has also been shown to inhibit RIG-I-TRIM25-mediated antiviral signaling pathway by direct interaction with MAVS (62, 63). Furthermore, this inhibition of type I and III IFNs was enhanced with Asn66Ser substitution. Intriguingly, PB1-F2 can also be expressed as a truncated (57 amino acids) protein devoid of the carboxyl terminus, which failed to translocate into the mitochondria (54). The full length PB1-F2 is mostly expressed by highly pathogenic viruses, such as H5N1, whereas low pathogenic subtypes, such as H1N1 (except PB1-F2 of 1918 H1N1) tend to express this truncated form. PB1-F2 also enhances the development of secondary bacterial pneumonia in mice with reduced survival rate. When PB1-F2 of A/PR/8 was engineered to become that of 1918 H1N1 by mutagenesis, the resulting mutant virus became more pathogenic with and without secondary bacterial infection (64). Surprisingly, this increased pathogenicity by PB1-F2 of 1918 H1N1 was also attributed to Asn66Ser (65). A mutant virus carrying H5N1 PB1-F2 gene with Asn66Ser in A/WSN/33 background led to increased pathogenicity with 100-fold increase in viral replication compared with wild-type virus in mice. Similarly, when the 1918 H1N1 virus was reconstructed to carry a pathogenicity-reducing substitution (Ser66Asn), the resulting virus led to attenuated viral replication and reduced mortality.

PB1-F2-mediated IAV pathogenicity could be the result of its interaction with NLRP3-inflammasome. PB1-F2 of H7N9 has been shown to form fibrillar higher molecular weight aggregates that were incorporated into the phagolysosome and induced ASC speck formation upon acidification (66). This resulted in the activation of NLRP3-mediated inflammasome, leading to increased production of pyrogenic cytokine IL-1β and enhance the pathogenesis of influenza viral pneumonia in mouse models (66, 67). PB1-F2 of avian H7N9 has also been shown to increase mitochondrial reactive oxygen species (ROS) and calcium (Ca^2+^) efflux, which contributed to the activation of NLRP3 inflammasome (68).

**Figure 3:**
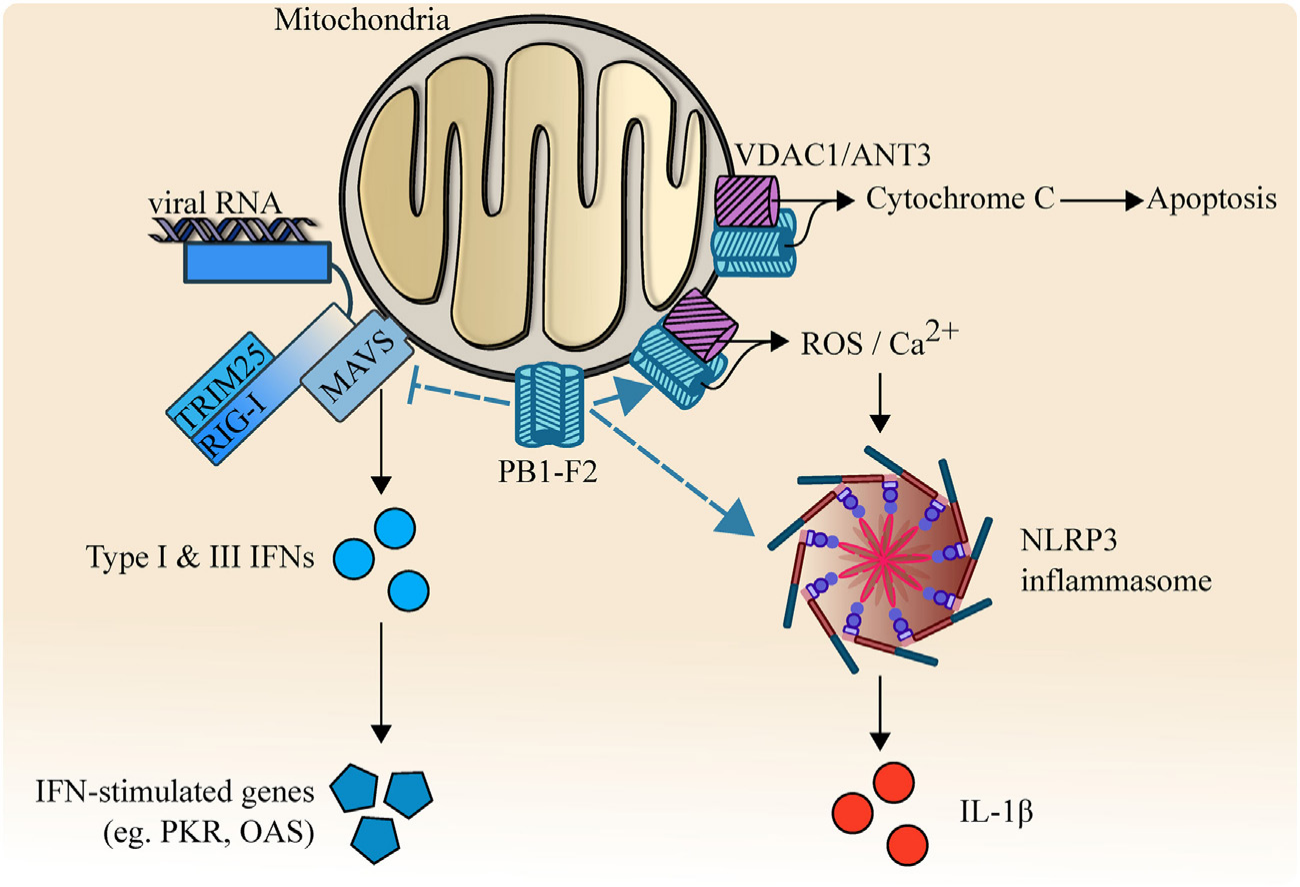
PB1-F2 and its multi-functional functions. PB1-F2 binds and inhibits RIG-I/TRIM25/MAVS-mediated signaling activity, reduces the transcription of type I, III interferon (IFNs), and the downstream induction of antiviral responses. PB1-F2 also forms fibrillar higher molecular weight aggregates that facilitate the release of pyrogenic IL-1β by activating NLRP3-dependent inflammasome. Outer mitochondrial membrane ion channel voltage-dependent anion channel 1 (VDAC1) and adenine nucleotide translocator 3 (ANT3) are also targeted by PB1-F2, leading to increased release of cytochrome C, reactive oxygen species (ROS), and Ca^2+^. These result in apoptosis and NLRP3-inflammasome activation.

PB1-F2, therefore, appears to be a major driving force for excessive pro-inflammatory cytokine storm and pathogenesis (Figure 3), and Asn66 is important in the function of PB1-F2. Nevertheless, how IAV-mediated inflammatory cytokine storm holds an advantage in IAV survival remains elusive. The formation of this fibrillary PB1-F2 aggregate is consistent with the crystal structural data that indicated a strong propensity for PB1-F2 to adopt an oligomerization structure and form membrane pores in planar lipid bilayers (69, 70). However, it remains unknown whether it is a requirement for PB1-F2 to form this fibrillar structure to interact with all of its binding targets, and while the oligomerization domain is located in carboxyl-terminal α-helix, it is unknown if oligomerization is dependent on Asn66. It is also unclear if this structure is unique to highly pathogenic strains or is present in all subtypes/strains.

## NS1—The Inverse Interferon

When IFNs were first described by Isaacs and Lindenmann, it was found that cells treated with heat-inactivated, but not live IAV could produce these antiviral cytokines, an immuno-inhibitory phenomenon he described as “inverse interference” (71, 72). The identity of Lindenmann’s inverse interferon was later revealed to be NS1 protein. NS1 protein is encoded by the NS gene together with nuclear export protein *via* alternative splicing. As NS1 is encoded by the shortest gene of all, NS1 protein is rapidly expressed to high levels in the infected epithelial cells (73, 74).

**Figure 4:**
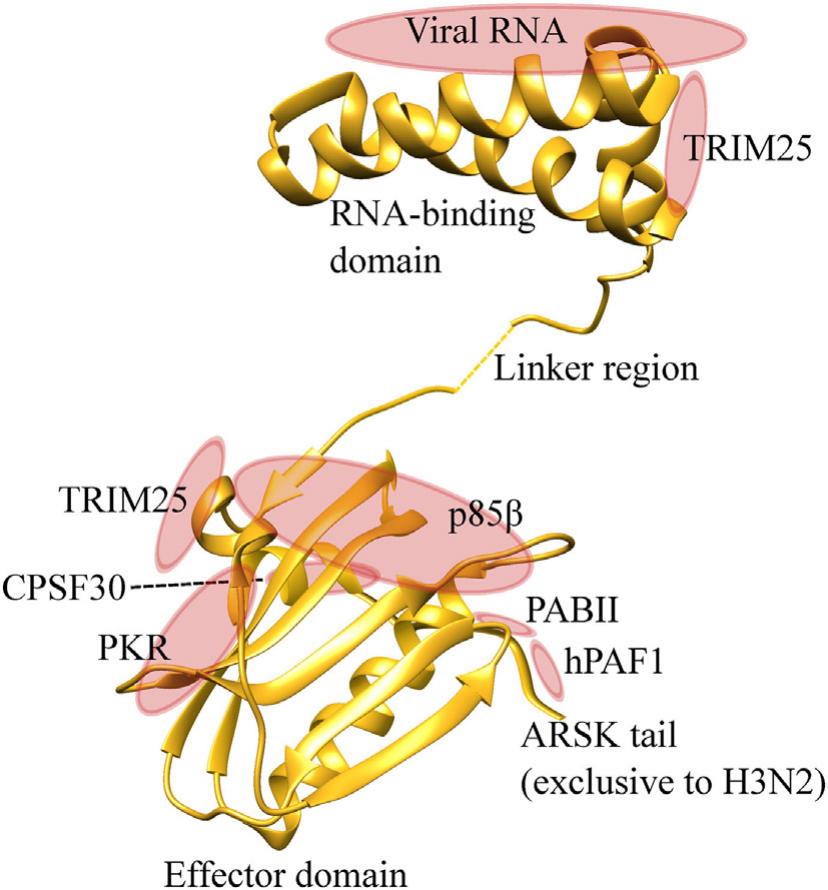
NS1 is an effective suppressor of host antiviral immunity. NS1 protein contains an amino-terminal RNA binding domain and carboxyl-terminal effector domain. RNA binding domain binds to viral RNAs and prevents detection by the host pattern recognition system. The effector domain binds to TRIM25, and inhibits RIG-I-MAVS interaction and downstream induction of type I and III IFNs. This domain also interacts with protein kinase R (PKR), therefore, inhibiting its pro-apoptotic activities. PI3K-p85β subunit is also targeted by the effector domain to inhibit apoptosis. NS1 binds with CPSF30 and poly A binding protein II (PABII), inhibiting host protein synthesis and facilitating viral protein synthesis. The ARSK tail is exclusively found in H3N2, and directly binds with hPAF1 complex and inhibits the transcription of antiviral genes. Protein visualization was performed in UCSF chimera molecular visualization application.

NS1 is a multi-functional protein with a RNA-binding domain at its amino-terminus (residue 1–73), and an effector domain (residue 74–230) at the carboxyl-terminus (73, 75, 76). The RNA-binding domain of NS1 recognizes dsRNA sequences and blocks the host RNA detection system. The effector domain of NS1 can stabilize the RNA-binding domain, but it predominantly interacts with host cellular proteins and interferes with host mRNA processing and host innate immune responses (Figure 4). NS1 inhibits host pre-mRNA endonucleolytic cleavage and polyadenylation by direct interaction and inhibition of cleavage and polyadenylation specificity factor −30 subunit (CPSF30) and poly A binding protein II (77–79). By shutting down the host cellular protein synthesis in the infected cells, this helps the virus to gain control of the host machineries required for efficient viral protein synthesis.

The major function of NS1 is to antagonize host innate immune responses during infection, and this occurs at multiple stages of the IFN signalling cascade. In the nucleus, NS1 of human IAV H3N2 was recently shown to contain carboxyl-terminal ARSK tail sequence (amino acids 226–229), which is analogous to the ARTK sequence on the lysine 4 of histone H3 (H3K4) (80). This ARSK tail could act as a H3K4 histone mimic that directly interacts with human polymerase II-associated factor 1 complex and impairs the transcription of antiviral genes. However, this ARSK tail appears to be unique to H3N2, and is absent in H5N1, H7N9, and H1N1pdm09. In the cytoplasmic space, NS1 inhibits RIG-I-mediated signaling and subsequent induction of type I IFNs (81, 82). Specifically, the NS1 protein inhibits RIG-I ubiquitination mediated by TRIM25, which is crucial for maximal type I and III IFNs expression during viral infection (83). This NS1-TRIM25 binding event is dependent on the Arg38 and Lys41 in the RNA binding domain, and Glu96 and Glu97 in the effector domain of NS1 (84, 85). NS1 protein also interferes with functions of important intracellular antiviral ISGs, including PKR and oligoadenylate synthase (OAS). The RNA-binding domain of NS1 can bind to viral RNA and avoid detection by PKR (86). It also binds to PKR itself via the effector domain (residue 123–127) and inhibits PKR-mediated viral mRNA suppression and apoptosis (87–90). OAS, which detects and cleaves viral RNA by activating RNase L, can also be blocked by influenza NS1. The RNA-binding domain of NS1 can out-compete the RNA binding capacity of OAS, thereby inhibiting the antiviral response (91). Another important target of NS1 is the PI3K signaling pathway. NS1 activates PI3K pathway by direct interaction with p85β subunit, thereby increasing the rate of viral internalization and inhibition of apoptosis. NS1-p85β interaction is dependent on Tyr89/Met93 (92), Leu141/Glu142 (93), and Pro164/Pro167 (94) of the effector domain, all of which are located adjacent to each other within a cleft between the two NS1 monomers. The anti-apoptotic effect of NS1-PKR and NS1-p85β interaction, however, appears to conflict with the pro-apoptotic function of PB1-F2. The molecular equipoise of pro- and anti-apoptotic response by these two contradicting signals and the timing of apoptosis remain elusive.

The carboxyl-terminus of the effector domain is essential in the NS1-mediated inhibition of antiviral responses. A mutant H7N7 virus that lacked NS1 gene or with a large carboxyl-terminus deletion showed impaired IFN suppression activity and attenuated replication in mammalian and avian epithelial cells (95). Similar findings were also observed with influenza viruses of different species. Both swine and turkey influenza virus with NS1 carboxyl-terminal truncation showed attenuated replication and an increase in IFN response compared to the wild type infection in its respective epithelial cells (96, 97). When the NS1 gene of high pathogenic avian H5N1 was replaced with the one that had a natural deletion of residues 191–195 from low pathogenic swine H5N1, the resulting virus was attenuated in viral replication and its IFNs inhibition in chickens (98). The deleted residues were then engineered into this NS1 and the resulting virus gained virulence, demonstrating the importance of these residues at 191–195 in IFN inhibition. The overall suppression of host antiviral immunity by the NS1 protein appears to be more effective with human IAV H3N2 compared with a low pathogenic subtype (39). This difference in the levels of inhibition by the NS1 proteins was likely due to differences in the amino acid residues discussed previously, which might be driven by the selective pressure and the long evolution of the virus in human populations. The H5N1 NS1 was able to dramatically reduce the induction of antiviral cytokines, leading to much higher viral replication (99, 100). Influenza NS1 is no doubt a powerful and fast-deployable antagonist that rapidly controls critical host immune infrastructure (Figures 3 and 4). The dual wields of NS1 and PB1-F2 thus allow for maximal viral replication with minimal interference from the host immune system, while causing severe disease in the infected individuals.

## Novel Therapeutic Strategies

Influenza vaccination remains the cornerstone of efforts to protect against influenza, however, there are serious concerns regarding current vaccine prediction and manufacturing process. Mis-match or unforeseeable mutations between the circulating strain and the vaccine strain often results in compromised herd immunity and increased infections in that year (101). Vaccine manufacturing is a slow and problematic process, and this issue became evident during the 2009 H1N1 pandemic. The first vaccines only became available 5 months after the identification of the virus, and mutations can occur during the vaccine manufacturing period, leading to reduced effectiveness than anticipated. Antiviral drugs, such as M2 channel and neuraminidase inhibitors, are mostly used as treatment for influenza. However, these drugs were mostly given to patients who had symptoms after the virus had shed, therefore, had limited effect. Neuraminidase inhibitors have been shown to be more effective in reducing length of hospital stay than a reduction of mortality rate (102, 103). Heavy use of these drugs has also resulted in a massive development of resistance, including the emergence of oseltamivir-resistant H1N1pdm09 (A/Newcastle/1/2011) in Newcastle, NSW, Australia (2). There is, therefore, an urgent need to identify and develop novel therapeutic targets for influenza, in preparation for future emergence of influenza viruses and pandemics.

The peptide-based therapeutics is an ideal approach that offers specificity with relatively minor side-effects, and can be administered directly to the lung *via* the pulmonary delivery route. This delivery route also increases the adsorption area in the lung by the therapeutic peptides. For these reasons, a number of peptides have been discovered to inhibit IAV replication at different stages of infection.

IAV HA represents an initial and critical step to a successful infection, and would be an ideal target for drug design as a preventative strategy. By screening a HA fragment peptide library, Chen and Guo identified a HA-targeting peptide, HA-pep25, that inhibited influenza virus binding and infection, including human H1N1, human and avian H5N1, and avian H7N9 (104). HA-pep25 is an 18-mer mimicking peptide that corresponds to the sialic acid residue binding site (HA_221–238_), and specifically binds to the sialic acid residues, thereby shielding the cells from the viral attachment. Jones et al. also demonstrated that a 20-amino acid peptide derived from fibroblast growth factor 4 was able to inhibit viral binding to sialic acid residues by several IAVs, including H1N1, H3N2, and H5N1 (105). Prophylactic treatment in mice substantially reduced viral replication, and post-infection treatment was also as effective as rimantadine in protection against H5N1 in mice. Other peptide-based inhibitors, such as Flupep and Flufirvitide, have also been shown to be effective in inhibiting viral attachment to the cells. Flupep interacts with HA and inhibits viral binding (H1N1pdm09, H3N2, and H5N1) to the cell membrane (106). Flufirvitide is a mixture of hydrophobic α-helical peptides that also binds with HA and blocks viral internalization and infection, and is currently being tested in clinical trials (107).

A class of synthetic anti-lipopolysaccharide peptides (SALPs) has been reported to also inhibit IAV attachment to cell surface. SALPs were originally designed to inhibit bacteria-mediated lethal septic shock in mice (108), but also possess antiviral activities against IAVs. Specifically, SALP PEP19-2.5 peptide showed high binding affinity toward the sialic acid residues, and led to reduced viral attachment and internalization, including human H3N2, H1N1pdm09, and high pathogenic H7N7 (109). Given that primary IAV infection is frequently followed by secondary bacterial infection, this class of peptides would be an ideal therapeutic approach for IAVs as a preventative or post-infection treatment. IAV polymerase complex is also an ideal therapeutic target due to its importance in viral protein synthesis in the host cells. PB1_1–25_ and PB1_715–740_ (110), and PB1_731–757_ (111) mimicking peptides have been separately shown to bind to a conserved PB1 binding site on PA subunit, and substantially reduced viral replication of a panel of viruses, including H1N1 and H5N1.

Advances in structural interactions between human broadly neutralizing antibodies and HA have directed several synthetic proteins and peptides that inhibit fusogenic conformational changes in the endosome. A panel of synthetic HA binder (HB) scaffold proteins have been shown to bind with the conserved region of the stems of HA protein (112, 113). HB36 and HB80 were shown to bind H1 and H5 HA stem with nanomolar affinity, and inhibited low-pH-induced conformational changes. HB80 also displayed similar levels of neutralization compared with the broadly neutralizing antibodies. Furthermore, Kadam et al. recently constructed a series of small cyclic peptides that resemble the epitopes of the stem-targeting broadly neutralizing antibodies (114). These peptidic fusion inhibitors bound to the conserved HA stem epitope at nanomolar affinity, and inhibited HA conformational change at low pH. Similar design strategies could also be applied to other conserved sites on HA, or to other virulence factors of IAV.

As PB1-F2 and NS1 are two major virulence factors that promote pro-inflammatory cytokine storm and inhibit important antiviral immunity, it is interesting to note that there has been no progress in identifying peptides that specifically target these two factors. Ideal therapeutic peptides need to either shield hosts from IAV internalization, or have anti-inflammatory properties without affecting or even increasing antiviral immune responses to infection. These peptides, therefore, are an attractive direction for therapeutics against IAVs, and may be combined with current antiviral drugs to increase the effectiveness of the anti-influenza treatment. The efficacy of peptide-based drugs as preventive agents, however, needs to be further explored, particularly in transmission models such as ferrets.

## Conclusion

The constant genetic mutations, recombination, and selection pressure ensure the survival of IAV and its continued threat to mankind. This rapid and threatening evolution of IAV enables the virus to infect multiple species, and with its superior immuno-modulatory tactics, the emergence of novel IAVs is unpredictable and inevitable.

HA is the key for the virus to infect, spread, and to create a pandemic. Understanding the absolute molecular requirements for IAV to cross species barriers and achieve sustainable human transmission, and/or aerosol-airborne transmissibility switch will be vital in the development of next-generation virus-targeted therapeutics. This is crucial as the potential scenario of future IAV pandemics is likely to start with a lack of an IAV-specific vaccine, and current antiviral drugs would be available, but with questionable beneficial effect. The efficacy of peptide-based antiviral drugs as preventative agents will be an important first line of defense. However, when infection occurs, inhibiting viral polymerases with specific anti-PB1/2/PA peptides may also be feasible.

The dynamic duet of PB1-F2 and NS1 effectively controls the host cellular machineries for efficient viral replication, while instigating severe cytokine storm and disarming the host innate antiviral immunity in the infected individuals. Neutralization of their functions would be desirable to reduce infection and potentially fatal symptoms. By the time the infected individuals are admitted to hospital with symptoms, the virus would have shed. Future therapeutics will, therefore, also need to be host-targeted to treat symptoms such as the exaggerated airway or systemic inflammation that is left behind by the virus.

Peptide-based therapeutics may be the new generation of antiviral drugs, and would be protective against multiple strains/subtypes of IAV if targeted at the essential and conserved regions. This together with ever-improving nanoparticle delivery technologies could be the future of drug design and intracellular delivery system, and must be explored in the calm before the storm.

The great 1918 influenza pandemic happened before, and it will happen again.

## Author Contributions

AH conceptualized and wrote the manuscript.

## Conflict of Interest Statement

The author declares that the research was conducted in the absence of any commercial or financial relationships that could be construed as a potential conflict of interest.
